# Brief education to increase uptake of influenza vaccine among pregnant women: a study protocol for a randomized controlled trial

**DOI:** 10.1186/1471-2393-14-19

**Published:** 2014-01-14

**Authors:** Valerie WY Wong, Daniel YT Fong, Marie Tarrant

**Affiliations:** 1 School of Nursing, Li Ka Shing Faculty of Medicine, University of Hong Kong, 21 Sassoon Road, Pokfulam, Hong Kong, China

**Keywords:** Influenza, Influenza vaccination, Antenatal, Prenatal, Pregnant women, Pregnancy, Education, Interventions, Randomised controlled trial

## Abstract

**Background:**

Pregnant women are the highest priority group for annual influenza vaccination. Studies have shown unacceptably low uptake of both seasonal and pandemic A/H1N1 influenza vaccination among pregnant women. This paper will describe the study protocol and methodology of a randomised controlled trial designed to assess the effectiveness of a brief educational intervention in improving the uptake of seasonal influenza vaccine among pregnant women in Hong Kong.

**Methods:**

A randomised controlled trial will be conducted with pregnant women in at least the second trimester of pregnancy from four publicly funded hospital antenatal clinics in Hong Kong. Participants will be randomly assigned to either one of the two treatment groups: standard care (control) or standard care plus brief education (intervention). Pregnant women in the standard care group will receive the usual antenatal care with an educational pamphlet developed by the Hong Kong Centre for Health Protection and those in the intervention group will be provided with usual care plus a brief ten-minute education intervention. Content of the education session will cover four core components recommended in the research literature. The primary study outcome will be the proportion of participants who have received influenza vaccine during their pregnancy. A total of 184 pregnant women (92 per group) will be required to give an 80% power to detect a treatment effect of 15%.

**Discussion:**

Most intervention studies aimed at improving influenza vaccination rates in pregnant women have targeted obstetric-care providers and the results of the two patient-oriented RCT interventions are conflicting. The high priority for vaccination given to pregnant women and the low influenza vaccination rate among pregnant women worldwide strongly indicates a need for interventions to improve uptake.

**Trial registration:**

This trial is registered with the Clinical Trials Registry at www.clinicaltrials.gov(NCT01772901).

## Background

Influenza is a threat to human life, global economies, and security in our increasingly interconnected world [1]. Due to its high attack rate and continuous antigenic drift, seasonal influenza causes 3 to 5 million cases of severe illness and 250,000 to 500,000 deaths every year [2]. In particular, the Influenza A virus has caused a number of severe pandemics over the past century. The Spanish flu pandemic in 1918 killed approximately 50 to 100 million people while the Asian and Hong Kong flus in 1957 and 1968 caused 4 to 8 million deaths in total [3,4]. In the recent 2009 A/H1N1 influenza pandemic it is estimated that more than 150,000 people died, and in contrast to typical influenza season, 80% were younger than 65 years of age [5]. In addition to significant influenza-related morbidity and mortality, there is a substantial socioeconomic cost attributable to influenza. Every year, influenza results in direct societal costs from absenteeism, lost productivity, related medical treatments, and excess hospitalization and indirect costs from preventive and control measures implemented by national governments such as vaccination programmes, school closure, and quarantine [6,7].

According to both the World Health Organization (WHO) and the Centers for Disease Control and Prevention (CDC), pregnant women, young children, especially those under two years of age, people with chronic illnesses, those aged 65 and above, and healthcare workers are all more susceptible to influenza infection [2,8]. Pregnant women have higher rates of influenza-related morbidity and mortality because of the immunologic and physiologic alterations that occur in pregnancy, and which affect the respiratory, cardiovascular, and other organ major systems [9,10]. During the 2009 A/H1N1 influenza pandemic [11], pregnant women contracting influenza were more likely to experience pregnancy complications such as emergency caesarean section, stillbirth, neonatal death, preterm birth [12-14], low birth weight [11,14,15], hospitalization [11,14,16], and maternal death [10,15,16]. Although the risk of influenza infection is lower in non-pandemic years, infected pregnant women still have a greater risk of influenza mortality, pregnancy complications and hospital admission [17-19]. They also have a higher risk of being hospitalized for longer duration [18]. The risk of hospitalization in pregnant women with influenza is equal to, or higher than, the rate in older adults aged 65 to 69 years and people with underlying chronic illnesses [20,21].

### Benefits of influenza vaccine

Influenza vaccine has been demonstrated to be both beneficial and safe for pregnant women and their foetus at all stages of pregnancy [22-24]. Influenza vaccine reduces both maternal complications from influenza and infant morbidity in the first six months of life. The recommendation to vaccinate pregnant women with influenza vaccine was originally proposed in 1960, ten years after the influenza vaccine was first developed [25]. Although the evidence on the safety and benefits of influenza vaccination during pregnancy was limited at the time, the US Surgeon General first recommended women to receive influenza vaccine during pregnancy to prevent flu-related complications and deaths [26]. Given the increasing evidence demonstrating the safety and immunogenicity of influenza vaccination in pregnancy and the maternal antibody transfer to the unborn infant, in 2005 the CDC recommended that all pregnant women receive trivalent inactivated influenza vaccine (TIV) [27]. In 2010, in the first randomised controlled trial, researchers administered influenza vaccine to pregnant women in Bangladesh who were in their third trimester [28]. The vaccine reduced the incidence of febrile respiratory illness in pregnant women by 36% and reduced the incidence of laboratory confirmed influenza in infants up to six months of age by 63%. Further observational studies have shown similar reductions in both maternal and infant morbidity and mortality [29,30].

In addition, studies have shown that influenza vaccine during pregnancy, even in the first trimester, poses no additional risk to the developing foetus. In a recent study of over 50,000 pregnant women in Denmark, of whom over 7,000 had received the 2009 A/H1N1 influenza vaccine, there was no excess risk of miscarriage or stillbirth among vaccinated women [31]. A smaller study of 323 vaccinated pregnant women and 1,329 matched control subjects also found no excess risk of spontaneous abortion or foetal malformations among participants receiving the 2009 A/H1N1 vaccine, irrespective of time of vaccination [32]. To date, no study has shown any increased risk of vaccine-related maternal complications or adverse foetal outcomes [24,33-35]. Given that a single vaccine can protect the pregnant women, the foetus and the infant, a 3-for-1 benefit, in 2012 the WHO [36], recommended that among high risk groups, pregnant women have the highest priority in seasonal influenza vaccination programmes. This recommendation was subsequently adopted by a number of other national governments [23,37-39].

### Influenza vaccine uptake rates

Despite the recommendations from the WHO and national governments, the uptake of influenza vaccine among pregnant women in most developed countries is still lower than uptake in general population, with rates ranging from 1.7% to 76.4% [40-49]. Limited studies have been conducted in Asia, mostly in Hong Kong. A pre-pandemic study by Lau et al. found that only 3.9% of pregnant women had been immunized with seasonal influenza vaccine [50]. Coverage during the 2009–10 A/H1N1 pandemic was 4.9% [51], while a recent population-based post-pandemic study during the 2010–11 influenza season found an uptake rate of 1.7% [48].

A number of studies have found that many pregnant women are still unaware that they should be vaccinated or have substantial concerns about adverse effects of the vaccine on them and/or their foetus [51-53]. In addition, many health-care providers (HCPs) do not routinely recommend that pregnant women be vaccinated as they themselves are often unaware of the recommendations or also share concerns about adverse effects of vaccination [54-58]. Furthermore, HCPs often hesitate to recommend vaccination to their pregnant clients, not because they believe the vaccine is actually unsafe during pregnancy [58], but likely because they perceive clients may blame the vaccine, and by extension them, for any negative pregnancy outcomes. Research has shown that pregnant women with more knowledge about the potential complications of influenza and of the safety of the influenza vaccine are more likely to be vaccinated [51,58,59].

### Strategies to improve influenza vaccination rates

To date however, few interventions have attempted to increase influenza vaccine uptake among pregnant women. Of the few interventions that have been conducted, most of the studies either have targeted healthcare professionals to encourage them to discuss influenza vaccine with pregnant women and to encourage pregnant women to take the vaccine or have involved multicomponent interventions as such the individual effect of patient-focused intervention cannot be evaluated [60-68]. Only three studies have investigated patient-targeted interventions, of which one used a historical control group and two were randomised controlled trials (RCTs). Yudin et al. found that an education intervention increased knowledge of influenza and vaccine rates from 19% (historical control) to 56% (post intervention) [68]. Furthermore, Meharry et al. evaluated the impact of providing an educational pamphlet with or without a statement verbalizing the benefit of influenza vaccine to both pregnant women and their infants from birth to six months of age and found that these measures increased vaccination rates from 46.9% (control) to 79% (educational pamphlet only) and 86.1% (educational pamphlet with verbalized benefit statement) [62]. However, Moniz et al. found that providing 12 weekly text messages about the importance of influenza vaccination in pregnancy did not significantly increase vaccination uptake [63]. Given the conflicting results from the few published studies, which were all conducted in North America, it is not clear if patient-oriented interventions can be effective in promoting influenza vaccination uptake among pregnant women, particularly in an Asian or Chinese population.

The low rate of vaccine uptake among this target group and the paucity of evaluated interventions both indicate a clear need to develop and test interventions to increase the low rate of influenza vaccine uptake among pregnant women. In addition, publicly-funded antenatal clinics in Hong Kong provide the majority of care to pregnant women but do not provide influenza vaccination. Therefore, pregnant women must obtain the vaccine from private settings, which are widely dispersed throughout the city. Thus, interventions targeting HCPs may not be effective in this and similar settings. Interventions targeting pregnant women themselves are more likely to be effective in increasing influenza vaccine uptake. If demonstrated to be effective, the proposed intervention could easily be adopted in various antenatal settings. Increasing the uptake of influenza vaccine among pregnant women could prevent unnecessary hospitalizations among pregnant women and newborn infants, both of whom are at greater risk of excess morbidity and mortality from influenza infection.

### Aims and hypotheses

The aim of this randomised controlled trial is to determine whether a brief educational intervention will increase uptake of influenza vaccine among pregnant women when compared with standard antenatal care. The primary study hypothesis is that the influenza vaccination rate will be increased among participants who receive the brief educational intervention when compared with those who receive standard antenatal care. The secondary study hypotheses are that participants in the intervention group will have a higher rate of self-initiated discussion about influenza vaccination with their healthcare provider, a higher rate of requesting to receive the influenza vaccine, and improved knowledge of and more positive attitudes toward influenza and influenza vaccine, when compared with those who receive standard antenatal care.

## Methods

This study will be an open-label, randomised controlled trial. Pregnant women in at least the second trimester of pregnancy will be recruited from the antenatal clinics of four public hospitals geographically dispersed throughout Hong Kong. Ethical approval has been obtained from the Institutional Review Boards of all the participating study sites and informed written consent will be obtained from participants who have agreed to participate but prior to randomisation. The design, implementation and reporting of this study will conform to the guidelines listed in the ICH-GCP, Declaration of Helsinki, standard CONSORT 2010 Statement [69].

### Study participants

Pregnant women attending the antenatal clinics of the four public hospitals will be screened for eligibility. Eligible patients are pregnant women: (a) with a singleton pregnancy; (b) at least 18 years of age; (c) in at least the second trimester of pregnancy; (d) Cantonese speaking; (e) Hong Kong resident; (f) without serious medical or obstetrical complications; (g) have not received the influenza vaccine in this pregnancy; and (h) who will be staying in Hong Kong for at least two weeks after birth.

### Randomisation

A research nurse will attend the antenatal clinics at the respective study sites and screen pregnant women for eligibility. Eligible women will be approached and informed about the study. Those who agree to participate will be given more in-depth information about the study details and informed written consent will be obtained. A third party, who will not be involved in any part of the study, will develop a computer-generated randomised treatment allocation sequence for each study site using random block sizes of two to eight. The treatment assignments will each be placed in sequentially numbered, opaque, sealed envelopes. After obtaining informed written consent, the research nurse will select the next envelope in the sequence to determine the treatment allocation. A research assistant, who will not be involved in participant recruitment and will be blinded to the participants’ treatment allocation, will be responsible for the assessment of the study outcomes.

### Study interventions

This trial compares standard antenatal care to a brief one-to-one, 10-minute education session by a research nurse explaining the facts of influenza and influenza vaccine and answering participant questions. Briefly, usual antenatal care consists of routine checking of the maternal and foetal health by either clinic midwives or obstetricians along with health education to promote a healthy pregnancy. Special childbirth preparation and breastfeeding classes are available to the pregnant women for no additional cost. Routine antenatal care does not usually encompass specific education or recommendations about influenza vaccination. However, we will provide the standard care group participants with an educational pamphlet on influenza vaccine in pregnancy developed by the Hong Kong Centre for Health Protection (CHP), which is freely available in all antenatal clinics.

The brief education group will receive the usual care plus one-to-one, brief education that will focus on four key recommendations identified from the research literature [52,60,70-73]: (i) inform pregnant women of vaccination recommendations, (ii) encourage discussion with their HCPs, (iii) increase accessibility of vaccine (make referral to clinics where vaccine can be obtained), and (iv) provide credible information from the government official website and provide the website URL. Specifically, for participants in the education group in this study we will inform them about the: (i) WHO and Hong Kong CHP recommendations regarding influenza vaccine during pregnancy, (ii) potential complications associated with influenza infection during pregnancy and for young infants, (iii) safety of influenza vaccine for pregnant women and the foetus, and (iv) potential benefits of influenza vaccine for the pregnant women, the foetus and the infant, and (iv) where and how to get the influenza vaccine in Hong Kong.

To ensure the consistency of intervention, one research nurse will implement the intervention across the four sites. The research nurse has completed extensive education on the international and national vaccination guidelines and the benefits and safety of influenza vaccination for pregnant women, as well as the implementation of randomised controlled trials.

### Outcome measures

The primary study outcome is the influenza vaccination rate during pregnancy in the control and intervention groups. The secondary study outcomes are the proportion of participants initiating discussion about influenza vaccine with their health care provider, the proportion of participants seeking out influenza vaccine, and the influenza and influenza vaccine knowledge of participants in the control and intervention groups after receiving the intervention.

### Data collection and measurements

After recruitment, pregnant women who agree to participate in the study and have signed a written consent form will be asked to complete a standard baseline questionnaire that will include (i) key demographic data (i.e., age, marital status, education level, family income, and employment status); (ii) maternal health data (i.e., pre-existing health conditions, pregnancy related health problems, gravidity and parity, and expected date of confinement), and (iii) influenza and influenza vaccine knowledge. Participants will be followed up by telephone at two to three weeks after their expected confinement date by a study research assistant who will be blinded to their treatment group allocation. During the follow-up telephone interviews, information will be collected on (i) influenza vaccination data (i.e., vaccination status, reasons for receiving or not receiving the vaccine, discussion with HCPs, attempts to receive vaccine, anti-vaccination advice from HCP or family members, and vaccination status of family members); (ii) health status during pregnancy (i.e., perceived health status during pregnancy, pregnancy-induced medical conditions, and respiratory illnesses and symptoms); (iii) actual birth data (i.e., date of birth, gender, gestational age and birth weight); and (iv) postnatal influenza and influenza vaccine knowledge.

### Sample size

Previous Hong Kong studies show that the range of seasonal influenza vaccine uptake among pregnant women ranges from 1.7 to 5.0% [48,50,51]. From the literature, studies have shown that with provider-focused interventions, influenza vaccine uptake rates increased from 1 to 2% before the intervention to approximately 37% after the intervention [64-66,68]. In addition, the Meharry et al. study evaluating the educational pamphlet plus the verbal statement increased vaccine uptake from 46.9% in the control group to 86.1% in the intervention group [62]. Thus, an estimate of the ‘normal’ prevalence of influenza vaccine uptake among pregnant women in Hong Kong would be 5.0% and an increase to 20% would be highly conservative but still clinically meaningful. Thus, using a study power of .80 and a significance level of .05, then 76 participants would be required for each group calculated using the Gpower z-test [74]. After accounting for a loss to follow-up or dropout rate of around 20%, approximately 92 participants per group are required for a total of 184 participants. Recruitment would begin once the influenza vaccine is available, usually early October, and continue until all participants are recruited.

### Statistical analysis

The primary analyses will compare the proportion of participants in the two study groups who have actually received the influenza vaccine during their pregnancy. We will assess the equivalency of treatment group allocation by comparing the major socio-demographic indicators (age, education, income). Between-group comparisons will be made using a chi-squared test. The intention-to-treat principle will be used with missing values taken as no vaccination. Each estimate will be accompanied by a 95% confidence interval and a 5% level of significance will be used in all statistical tests. Other data analysis will include comparing the proportion of participants initiating discussion of influenza vaccine with their health-care provider and the proportion of participants seeking out influenza vaccination, again using chi-square tests. The knowledge level of the participants will be compared before and after the intervention using chi-squared test and student’s t-test. Data analysis will be performed using the Stata version 13.1 statistical software [75].

## Discussion

The current body of evidence does not provide sufficient guidance on the effectiveness of patient-oriented interventions in promoting influenza vaccination uptake among pregnant women. The comprehensive intervention protocols with structured content targeting individual pregnant women increase the consistency of intervention delivery and adherence to study protocols. The multiple outcomes will provide valuable data to measure the impact of interventions. If brief education is shown to be effective in improving influenza vaccine uptake, it can be a low cost strategy to improve the low vaccination uptake among pregnant women, which can be easily adopted in various antenatal settings. Further studies can also be carried out to investigate the feasibility of shortening the brief education and also partnering it with providing on-site vaccination to maximize the potential benefit. Brief education can also be a cost-effective intervention, as increased influenza vaccination uptake would prevent unnecessary influenza-related hospitalization, excess morbidity and mortality among pregnant women and young infants.

### Ethical approval

Ethical approval for this study was obtained from (1) the Institutional Review Board of the University of Hong Kong/ Hospital Authority Hong Kong West Cluster (HKU/HA HKW IRB) (Ref No. UW 12–439), (2) Kowloon West Cluster Research Ethics Committee (KWC-REC) (Ref.#KW/EX/13-075), and (3) the Ethics Committee of Hong Kong East Cluster (EC-HKEC) (Ref.#HKEC-2013-072).

## Competing interest

The authors declare that they have no conflicts of interest to report.

## Authors’ contributions

VW contributed to the study design, delivered the intervention and wrote the first draft of the manuscript. DF contributed to the study design and critically reviewed and revised the manuscript. MT conceptualized the study, obtained funding, oversaw the implementation of the study, and critically reviewed and revised the manuscript. All authors have reviewed and approved the final version of this manuscript.

## Pre-publication history

The pre-publication history for this paper can be accessed here:

http://www.biomedcentral.com/1471-2393/14/19/prepub
